# A Novel Prognostic Scoring System Integrating Gene Expressions and Clinicopathological Characteristics to Predict Very Early Relapse in Node-Negative Estrogen Receptor-Positive/HER2-Negative Breast Cancer

**DOI:** 10.3389/fonc.2020.01335

**Published:** 2020-09-11

**Authors:** Caijin Lin, Jiayi Wu, Lin Lin, Xiaochun Fei, Xiaosong Chen, Ou Huang, Jianrong He, Weiguo Chen, Yafen Li, Kunwei Shen, Li Zhu

**Affiliations:** ^1^Department of General Surgery, Comprehensive Breast Health Center, Ruijin Hospital, Shanghai Jiao Tong University School of Medicine, Shanghai, China; ^2^Department of Clinical Laboratory, Ruijin Hospital, Shanghai Jiao Tong University School of Medicine, Shanghai, China; ^3^Department of Pathology, Ruijin Hospital, Shanghai Jiao Tong University School of Medicine, Shanghai, China

**Keywords:** breast neoplasm, first-2-year relapse, endocrine response, prognosis, model development

## Abstract

**Background:** Despite low aggressiveness in tumor biology and high responsiveness to endocrine therapy, subgroups of patients with estrogen receptor-positive/HER2-negative (ER+/HER2-) breast cancer relapse early in the first two years after initiation of endocrine therapy, indicating potential endocrine resistance. Accordingly, we attempted to establish a scoring system to inform the first-2-year prognosis (F2P Score).

**Methods:** Patients with node-negative ER+/HER2- breast cancer and complete data of gene expressions in a 21-gene panel were retrospectively retrieved from Shanghai Jiao Tong University Breast Cancer Database (SJTU-BCDB). The F2P Score was established based on the clinical and genomic variables associated with the first-2-year relapse after shrinkage correction and validated using the bootstrap resampling method. Model performance was quantified by Harrell's concordance-index (C-index) and Bayesian information criteria (BIC).

**Results:** The F2P Score was established by integrating the clinical (age and tumor size) and genomic (*ESR1, PGR, BCL2, CD68, GSTM1*, and *BAG1*) variables with a C-index of 0.71 and BIC of 397.46. Bootstrap C-index was 0.72 (95% CI, 0.62–0.81) and BIC was 396.75 (95% CI, 252.37–541.13). A higher score indicated an increased likelihood of a first-2-year relapse, when used as continuous (HR, 2.94; 95% CI, 1.87–4.61) or categorical (HR, 3.68; 95% CI, 1.70–8.00) predictors in multivariate analysis. Both continuous and categorical F2P Score also remained prognostic for overall survival and other endpoints. No significant interaction was observed between the F2P Score and treatment subgroups. Additionally, the F2P Score outperformed the IHC4, clinical treatment score and 21-gene test in predicting first-2-year relapse.

**Conclusion:** The F2P Score reported herein, integrating the clinicopathological and genomic variables, may inform prognosis and endocrine responsiveness. After the benefits and risks have been considered, treatment escalation may be an alternative strategy for patients with a higher score.

## Introduction

Estrogen receptor-positive and human epidermal growth factor receptor 2-negative (ER+/HER2–) breast cancer constitutes ~70% of malignant breast neoplasms (1, 2). Endocrine therapy is considered the therapeutic backbone for this subtype of breast cancer by counteracting estrogen-promoted tumor growth (1). Despite high endocrine responsiveness, there is a persistent risk of relapse in years 0–20 for ER+ breast cancer, and 5 to 10% of patients relapse early in the first two years after the initiation of endocrine therapy (3–5).

Early relapse during the first two years of endocrine therapy usually indicates the high aggressiveness of tumor biology and potential resistance to endocrine therapy, which remains one of the leading causes of treatment failure (6, 7). Some headway has been made concerning the underlying mechanisms of endocrine resistance, including the mutations in the ligand-binding domain of *ESR1*, the downregulation of progesterone receptor (PR) by hyperactive crosstalk between ER and growth factor signaling pathways, and the imbalance between the non-apoptotic and pro-apoptotic functions of *BCL2* family (8–12). These studies reinforce the idea that molecular biomarkers alone cannot yield accurate predictions for endocrine sensitivity and the likelihood of early relapse. From a clinical perspective, it is of great importance to develop a prognostic approach for relapse in the first two years, since treatment escalation is required for patients classified as high risk of very early relapse who are potentially endocrine-resistant.

To date, several multigene assays have been validated to estimate prognosis for the first five years (5, 13–16). Yet, the inferior prognostic capability of these assays was reported when compared to their combination with conventional clinicopathological factors (17–19). Additionally, it remains unclear that if these genomic assays allow the dichotomization of patients at high risk of first-2-year relapse. To address the issue, we attempted to build a scoring system that integrated the clinicopathological factors and gene expressions derived from a 21-gene panel for assessing the first-2-year prognosis (F2P Score) and informing the endocrine responsiveness.

## Materials and Methods

### Patients Selection

Women with histologically confirmed invasive breast cancer from 2009 to 2016 were retrospectively selected from the Shanghai Jiao Tong University Breast Cancer Database (SJTU-BCDB). Patients were included based on the following criteria: (1) immunohistochemically (IHC) determined ER positivity with ≥1% immunoreactive tumor cell nuclei (20); (2) HER2 negativity if scored 0/1+ by IHC or 2+ with non-amplified *HER2* gene being found on fluorescence *in situ* hybridization (HER2/CEP17 ratio <2.0 with average *HER2* gene copy number <6.0 signals/cell, or average *HER2* gene copy number <4.0 signals/cell regardless of the ratio) (21); (3) no lymph node involvement; (4) available reports of a 21-gene test. We excluded patients with incomplete clinicopathological characteristics and follow-up data, those diagnosed with *de novo* metastatic breast cancer, and those who had received neoadjuvant systemic therapy.

### Variables Defining

Clinicopathological variables used in the following analyses included age, marital status, menopausal status, comorbidity score, histology, grade, tumor size, PR status, and Ki67. Of these variables, comorbidity scores were calculated based on the sum of a series of comorbid conditions (each was assigned a score of 1, 2, 3, or 6), and then categorized into 0, 1, and ≥2 (22, 23). Clinical treatment score (CTS) and IHC4, proposed by Cuzick et al., were used in the procedure of model comparison (24). CTS was computed using age, tumor size, node status, grade, and use of anastrozole, while IHC4 was calculated based on ER, PR, HER2, and Ki67.

### Expression of Genes in the 21-Gene Panel

As was reported in our previous study, the expression of the 16 cancer-related genes was measured based on the 21-gene recurrence score assay (25). The tests were performed using formalin-fixed, paraffin-embedded tissue as previously described (5). First, hematoxylin and eosin-stained slides were reviewed to ensure sufficient tissue of invasive breast cancer by a pathologist, and then deparaffinization of the two 10 μm unstained sections was performed using xylene followed by ethanol. RNA extraction was performed using the RNeasy FFPE kit (QIAGEN, Hilden, Germany). Total RNA content was quantified, and the absence of DNA contamination was confirmed. After that, we conducted gene-specific reverse transcription followed by standardized quantitative reverse transcriptase-polymerase chain reactions (RT-PCR) in 96-well plates with Applied Biosystems (Foster City, CA) 7500 Real-Time PCR system. The PCR cycling went as follows: 95°C for 10 min for one cycle, 95°C for 20 s, and 60°C for 45 s for 40 cycles. The expression of each gene was measured in triplicate and normalized relative to five reference genes. ΔCT was computed as the mean CT value of the reference minus the CT value of the targeted cancer-related genes. The recurrence score was derived from the reference-normalized expression measurement for the 16 cancer-related genes (5).

### Statistical Analysis

The primary endpoint was 2-year invasive disease-free survival (IDFS). Secondary endpoints included 2-year distant disease-free survival (DDFS), distant relapse-free survival (DRFS, excluding death from any causes), and overall survival (OS). Detailed definitions were described by the STEEP system (26).

Cox proportional hazard model was developed to estimate the regression coefficients, hazard ratios (HR), and 95% confidence intervals (CI) for the clinical and genomic variables associated with first-2-year relapse. In this procedure, variables with a two-sided *P* < 0.1 were selected to establish the scoring system. To improve the predictive value and allow for overfitting, we estimated the global shrinkage factors to penalize the regression coefficients of the clinicopathological and genomic variables, respectively (24, 27). After that, the F2P Score was established based on the following equations, where the η denoted the shrinkage factors, β_*i*_ and β_*i*_' referred to the corresponding regression coefficients of the clinical and genomic variables, *v*_*i*_ were clinical variables (continuous or categorical), and Δ*CT*_*i*_ were computed as described in section Variables Defining:

$$\begin{array}{l} \text{F}2\text{P Score}={\text{η}}_{\text{clinical}}\times \left({\text{β}}_{1}\times {\text{v}}_{1}+{\text{β}}_{2}\times {\text{v}}_{2}+\ldots +{\text{β}}_{\text{n}}\times {\text{v}}_{\text{n}}\right)\;+\\ {\text{ η}}_{\text{genomic}}\times \left({\text{β}}_{1}\text{'}\times \text{ΔC}{\text{T}}_{1}+{\text{β}}_{2}\text{'}\times \text{ΔC}{\text{T}}_{2}+\ldots +{\text{β}}_{\text{n}}\text{'}\times \text{ΔC}{\text{T}}_{\text{n}}\right) \end{array}$$

After that, the F2P Score was internally validated using the bootstrap resampling method with 1,000 resamples.

The performance of the F2P score was quantified and compared using Harrell's concordance index (C-index), Bayesian information criteria (BIC), and the change in likelihood ratio χ^2^ (ΔLR-χ^2^). In our study, the net reclassification index (NRI) was also adopted to assess the reclassification performance and improvement of the model (28). When the baseline and new models were nested, NRI>0 indicates the improved performance of the new model. Also, the continuous relationship between the F2P Score and log-hazard ratio of first-2-year relapse was presented by cubic smoothing spline approximation. To assess the performance of the scoring system as a categorical predictor, the incidence of first-2-year relapse was estimated using the Kaplan-Meier method and compared using the Log-rank test, with the optimal cutoff point determined by X-tile (version 3.6.1; Yale University, New Haven, CT, USA).

In exploratory analyses, both the landmark analyses with a landmark point in the second year and the tests for interaction between time (0–2- vs. 2–5-years) and clinical/genomic variables were performed to explore the time-dependent effect on relapse. All tests adopted two-tailed *P* < 0.05 suggesting statistical significance unless otherwise stated. *Survival* package (version 3.1-8) was used for performing the Kaplan-Meier method, Cox proportional hazards model, and landmark analysis, *shrink* package (version 1.2.1) for the calculation of shrinkage factors, *boot* package (version 1.3-24) for bootstrap resampling method, and *nricens* package (version 1.6) for the calculation of NRI. All statistical analyses were performed in R version 3.5.3 (www.r-project.com).

## Results

### Baseline Characteristics

Detailed clinicopathological characteristics and distribution of endpoint events were summarized in Table 1. A total of 1,156 patients were identified. Thirty in 66 (45.5%) IDFS events and 10 in 21 (47.6%) distant relapses were observed for the first two years, and the annual rates were 1.39 and 0.47%, respectively. When compared to those without first-2-year relapse, worse prognosis was observed for patients who relapsed on the first two years (log-rank *P* < 0.001), with a 5-year OS of 98.8% (95% CI 97.8–99.8%) and 70.6% (95% CI 55.1–90.5%), respectively (Figure S1).

**Table 1:** Clinicopathological characteristics (*N* = 1,156).

**Characteristics**		**Number of patients (%)**
**Age, years**		
≤ 50		361 (31.2)
>50		795 (68.8)
**Marital status**		
Married		1105 (95.6)
Unmarried		51 (4.4)
**Menopausal status**		
Postmenopausal		729 (63.1)
Premenopausal		427 (36.9)
**Comorbidity score**		
0		946 (81.8)
1		120 (10.4)
≥2		90 (7.8)
**Histology**		
IDC		991 (85.7)
ILC		47 (4.1)
Others		118 (10.2)
**Grade**		
Low		113 (9.8)
Intermediate		642 (55.5)
High		240 (20.8)
Unknown		161 (13.9)
**Tumor size, cm**		
Continuous^a^		1.8 (1.3–2.5)
≤ 2		812 (70.2)
>2		344 (29.8)
**PR**		
Negative		172 (14.9)
Positive		984 (85.1)
**Ki67**		
<20%		692 (59.9)
≥20%		464 (40.1)
**Endocrine therapy**		
TAM		470 (40.7)
AI		686 (59.3)
**Ovarian suppression**^**b**^		
Yes		49 (11.5)
No		378 (88.5)
**Chemotherapy**		
Yes		557 (48.2)
No		599 (51.8)
**Radiotherapy**		
Yes		501 (43.3)
No		655 (56.7)
**All events**		
0–5-years	No. of events	66
	Events/y (%)	1.71
0–2-years	No. of events	30
	Events/y (%)	1.39
2–5-years	No. of events	36
	Events/y (%)	1.93
**Distant relapse**		
0–5-years	No. of relapse	21
	Relapse/y (%)	0.65
0–2-years	No. of relapse	10
	Relapse/y (%)	0.47
2–5-years	No. of relapse	11
	Relapse/y (%)	0.77

IDC, infiltrating ductal carcinoma; ILC, infiltrating lobular carcinoma; PR, progesterone receptor.

a Data were presented as the median value with the interquartile range in parenthesis.

b *Only premenopausal women were presented*.

### Association Between Clinical and Genomic Variables and First-2-Year Relapse

In univariate Cox regression analyses, greater tumor size was associated with increased risk of first-2-year relapse (HR 4.15, 95% CI 1.98–8.72, *P* < 0.001), and age >50 years (HR 0.51, 95% CI 0.25–1.05, *P* = 0.068) and PR-negativity (HR 2.06, 95% CI 0.92–4.62, *P* = 0.081) also trended toward significance (Table 2). No significant interaction between time periods and clinical variables was observed (Table S1).

**Table 2:** Cox regression analysis revealing the association of clinicopathological and genomic variables with first-2-year relapse.

	**Coefficient**	**HR**	**95% CI**	***P***
**Part I: Clinical variables**				
Age, vs. ≤ 50-years	−0.667	0.51	0.25–1.05	0.068
Menopausal status, vs. postmenopausal	0.265	1.30	0.63–2.68	0.472
**Comorbidity score**				
1 vs. 0	0.412	1.51	0.52–4.40	0.450
≥2 vs. 0	0.943	2.57	0.97–6.81	0.058
**Histology**				
ILC vs. IDC	−0.137	0.87	0.12–6.44	0.893
Others vs. IDC	0.600	1.82	0.70–4.78	0.222
**Grade**				
Intermediate vs. low	0.916	2.50	0.33–19.01	0.376
High vs. low	1.328	3.77	0.47–30.18	0.211
Tumor size, vs. ≤ 2 cm	1.424	4.15	1.98–8.72	<0.001
PR status, vs. PR-positive	0.721	2.06	0.92–4.62	0.081
Ki 67, vs. <20%	0.134	1.14	0.56–2.35	0.717
**Part II: Genomic variables**				
**Estrogen module**				
ER	−0.259	0.77	0.62–0.96	0.017
PGR	−0.191	0.83	0.71–0.96	0.015
BCL2	−0.292	0.75	0.54–1.03	0.075
SCUBE2	−0.075	0.93	0.77–1.12	0.445
**Proliferation module**				
Ki67	0.302	1.35	0.96–1.89	0.102
STK15	0.045	1.05	0.78–1.40	0.761
Survivin	0.095	1.10	0.82–1.48	0.532
CCNB1	−0.169	0.85	0.61–1.17	0.309
MYBL2	0.108	1.11	0.82–1.51	0.489
**Invasion module**				
MMP11	−0.018	0.98	0.77–1.25	0.884
CTSL2	0.165	1.18	0.88–1.58	0.264
**HER2 module**				
GRB7	−0.150	0.86	0.60–1.23	0.414
HER2	−0.132	0.88	0.64–1.20	0.404
GSTM1	−0.226	0.80	0.59–1.06	0.092
CD68	−0.457	0.63	0.46–0.88	0.006
BAG1	−0.320	0.73	0.51–1.04	0.081

*IDC, infiltrating ductal carcinoma; ILC, infiltrating lobular carcinoma; PR, progesterone receptor; HR, hazard ratio; CI, confidence interval*.

**Figure 1:**
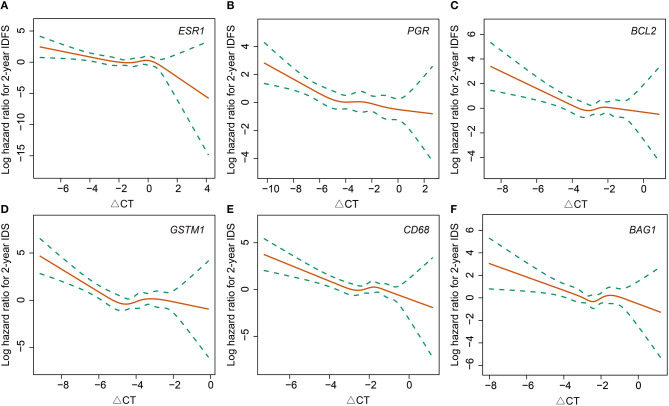
Association between 2-year invasive disease-free survival and gene expressions of ESR1 **(A)**, PGR **(B)**, BCL2 **(C)**, GSTM1 **(D)**, CD68 **(E)**, and BAG1 **(F)**. IDFS, invasive disease-free survival.

In terms of gene expressions, increased expression of *ESR1* (HR 0.77, 95% CI 0.62–0.96, *P* = 0.017), *PGR* (HR 0.83, 95% CI 0.71–0.96, *P* = 0.015), and *CD68* (HR 0.63, 95% CI 0.46–0.88, *P* = 0.006) proved to be predictors for lower risk of first-2-year relapse. Likewise, the trend was also observed with increased expression of *BCL2* (HR 0.75, 95% CI 0.54–1.03, *P* = 0.075), *GSTM1* (HR 0.80, 95% CI 0.59–1.06, *P* = 0.092), and *BAG1* (HR 0.73, 95% CI 0.51–1.04, *P* = 0.081) (Table 2 and Figures 1A–F). Interestingly, the opposite directions of HRs for *PGR, CD68*, and *BAG1* were presented between two time periods (0–2- vs. 2–5-years) with interaction *P*-values of 0.033, 0.002, and 0.024, respectively. The expression of *ESR1* also presented such a trend (interaction *P* = 0.071) (Table S1).

### Model Development, Comparison, and Validation

Although a *P*-value of 0.081 was observed, PR status was not selected for model development due to the correlation between *PGR* expression and PR status. To avoid collinearity and improve the predictive accuracy, *PGR* expression was finally selected for the model development due to a lower *P*-value of 0.015. For the comorbidity score, a three-level variable, the overall *P*-value was 0.150, and thus, it was not selected as well. Consequently, the F2P Score was established based on the combination of six genomic variables (*ESR1, PGR, CD68, BAG1, BCL2*, and *GSTM1*) and two clinicopathological variables (age and categorical tumor size) with the shrinkage factors of 0.314 and 0.888, respectively. The formula of the F2P Score was developed and presented herein: F2P Score = 0.888 × (1.424 × tumor size-0.667 × age)+0.314 × (−0.259 × *ESR1*−0.191 × *PGR*−0.292 × *BCL2*−0.226 × *GSTM1* −0.457 × CD68–0.320 × *BAG1*).

Model performance of F2P Score was evaluated with C-index of 0.71 and BIC of 397.46. Compared with other variables, the prognostic performance of the F2P Score was superior to age (C-index, 0.58; BIC, 419.98; ΔLR-χ^2^ −22.52; NRI −0.52, 95% CI −0.88 to −0.05), tumor size (C-index, 0.66; BIC, 408.43; ΔLR-χ^2^ −10.97; NRI −0.05, 95% CI −0.66–0.38), and six-gene model (C-index, 0.59; BIC, 414.62; ΔLR-χ^2^ −17.16; NRI −0.70, 95% CI −1.04 to −0.23) (Table 3). When internally validated by the bootstrap resampling method, a stable performance was observed for the F2P Score with the C-index of 0.72 (95% CI, 0.62–0.81) and BIC of 396.75 (95% CI, 252.37–541.13) (Table 3).

**Table 3:** Performance of the models.

	**C-index**	**BR C-index**	**BIC**	**BR BIC**	**ΔLR-χ^2^**	***P***	**NRI (95% CI)**
**Performance of each model**							
F2P Score	0.71	0.71(0.61–0.80)	397.46	396.75 (252.37–541.13)	Reference	–	Reference
Age	0.58	0.58 (0.49–0.66)	419.98	419.34 (268.91–569.76)	−22.52	<0.001	−0.52 (−0.88 to −0.05)
Tumor size	0.66	0.67 (0.58–0.76)	408.43	407.66 (260.67–554.66)	−10.97	<0.001	−0.05 (−0.66–0.38)
Six–gene model	0.59	0.59 (0.49–0.70)	414.62	413.42 (264.78–562.06)	−17.16	<0.001	−0.70 (−1.04 to −0.23)
**Comparison of the performance between F2P Score and IHC4**							
IHC4	0.54	0.56 (0.47–0.64)	422.68	422.88 (271.69–574.06)	Reference	–	Reference
F2P Score	0.71	0.71(0.61–0.80)	397.46	396.75 (252.37–541.13)	25.22	<0.001	0.60 (0.14–0.98)
**Comparison of the performance between F2P Score and clinical treatment score**							
CTS	0.62	0.62 (0.50–0.74)	314.36	315.80 (192.13–439.47)	Reference	–	Reference
F2P Score	0.68	0.65 (0.54–0.76)	303.17	302.77 (176.92–428.63)	11.19	<0.001	0.36 (−0.37–0.86)
**Comparison of the performance between F2P Score and 21-gene recurrence score**							
RS	0.61	0.61 (0.50–0.71)	417.68	417.85 (268.47–567.23)	Reference	–	Reference
F2P Score	0.71	0.71(0.61–0.80)	397.46	396.75 (252.37–541.13)	20.22	<0.001	0.62 (0.22–1.00)

BR, bootstrap resampling; BIC, Bayesian information criteria; NRI, net reclassification index; CI, confidence interval; LR, likelihood ratio; RS, recurrence score.

*F2P Score is established based on the combination of six genomic variables (ER, PGR, CD68, BAG1, BCL2, and GSTM1) and two clinical variables (age and categorical tumor size). Six-gene model is established based on the six genomic variables only (ER, PGR, CD68, BAG1, BCL2, and GSTM1). The comparison between F2P Score and CTS was performed in patients with known grade and thus, different C-index and BIC of F2P Score was observed*.

Additionally, the F2P score also worked better in predicting first-2-year relapse when compared to IHC4 (C-index 0.54; BIC 422.68), with ΔLR-χ^2^ of 25.22, and NRI of 0.60 (95% CI 0.14–0.98). Similar results were observed when compared to clinical treatment score (C-index 0.62; BIC 314.36) with ΔLR-χ^2^ of 11.19 and NRI of 0.36 (95% CI −0.37–0.86), and 21-gene recurrence score (C-index 0.61; BIC 417.68) with ΔLR-χ^2^ of 20.22, and NRI of 0.62 (95% CI 0.22–1.00) (Table 3). Stable and consistent results were revealed after adopting the bootstrap resampling method.

### Association Between F2P Score and First-2-Year Relapse

**Figure 2:**
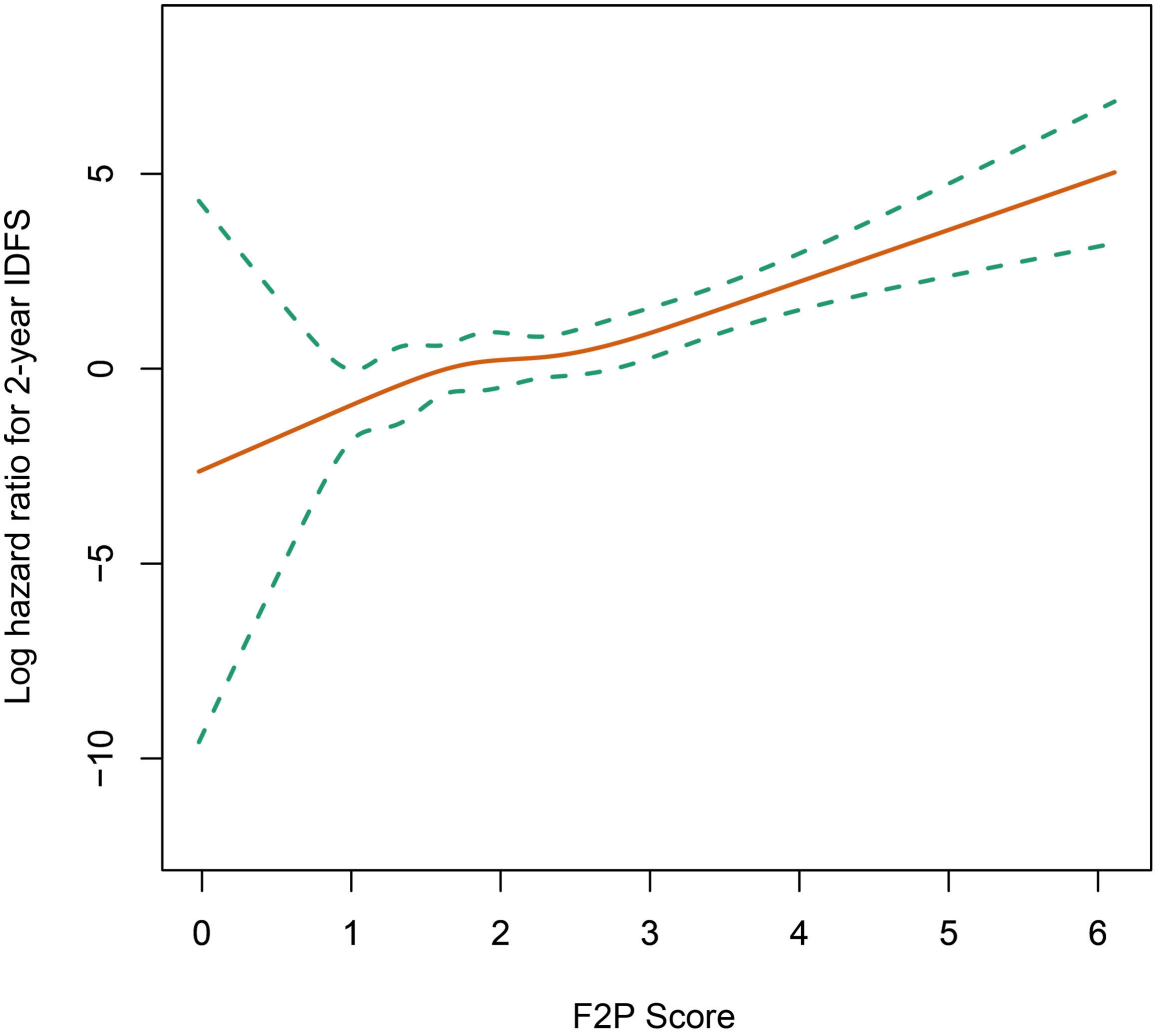
Association of F2P Score with 2-year invasive disease-free survival. IDFS, invasive disease-free survival.

**Figure 3:**
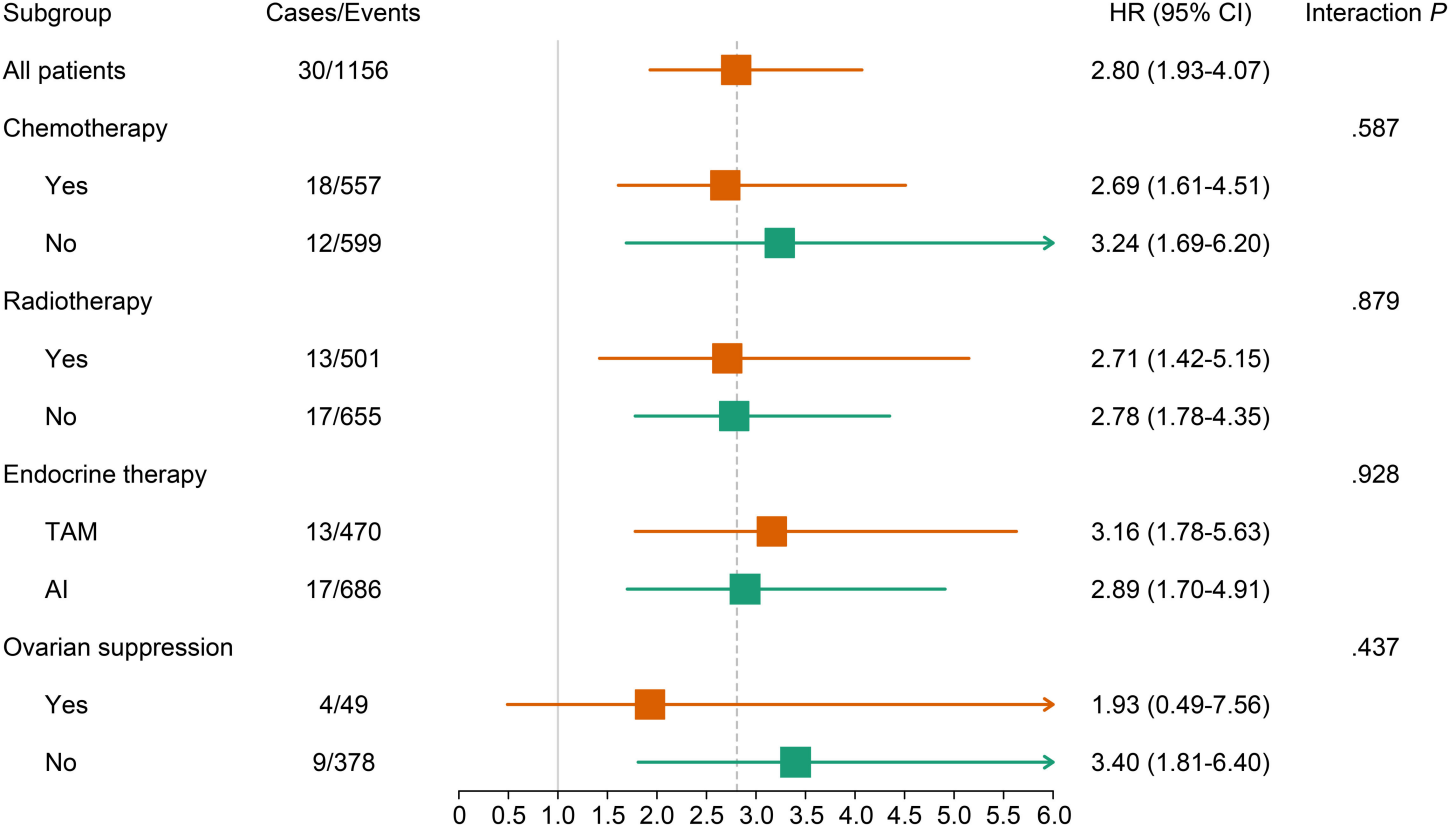
Association of continuous F2P Score with 2-year invasive disease-free survival across treatment subgroups. The subgroup analysis regarding the ovarian suppression was performed in premenopausal women. AI, aromatase inhibitor; CI, confidence interval; TAM, tamoxifen.

A continuously increasing association was observed between the F2P score and the predicted risk of first-2-year relapse (Figure 2) with significant interaction between two periods of years 0–2 vs. 2–5 (interaction *P* = 0.003). A higher score was indicative of an increased likelihood of first-2-year relapse both before (HR, 2.80; 95% CI 1.93–4.07; *P* < 0.001) and after (HR, 2.94; 95% CI, 1.87–4.61; *P* < 0.001) the adjustment for clinicopathological parameters (Table 4). Subgroup analysis revealed no substantial heterogeneity regarding the prognostic ability across the treatment subgroups (all interaction *P* > 0.05) (Figure 3).

**Table 4:** Association of the F2P Score with different end points.

	**Continuous F2P Score**		**Categorical F2P Score**	
	**HR (95% CI)**	***P***	**HR (95% CI)**	***P***
**Invasive disease-free survival (STEEP definition)**				
Univariate analysis	2.80 (1.93–4.07)	<0.001	4.07 (1.97–8.37)	<0.001
Multivariate analysis	2.94 (1.87–4.61)	<0.001	3.68 (1.70–8.00)	0.001
**Invasive disease-free survival (excluding second primary non-breast cancer)**				
Univariate analysis	3.40 (2.29–5.05)	<0.001	5.55 (2.45–12.55)	<0.001
Multivariate analysis	2.02 (1.44–2.82)	<0.001	5.37 (2.24–12.92)	<0.001
**Distant disease-free survival (STEEP definition)**				
Univariate analysis	3.83 (2.45–5.97)	<0.001	9.32 (3.01–28.90)	<0.001
Multivariate analysis	4.13 (2.28–7.49)	<0.001	8.09 (2.43–26.94)	<0.001
**Distant relapse-free survival (excluding all-cause mortality)**				
Univariate analysis	3.50 (1.94–6.30)	<0.001	7.26 (1.88–28.07)	0.004
Multivariate analysis	2.93 (1.35–6.35)	0.007	3.60 (0.85–15.34)	0.083
**Overall survival (STEEP definition)**				
Univariate analysis	3.42 (1.91–6.12)	<0.001	7.71 (1.50–39.76)	0.015
Multivariate analysis	6.01 (1.94–18.65)	0.002	11.97 (2.07–69.21)	0.006

STEEP, Standardized definitions for efficacy end points; HR, hazard ratio; CI, confidence interval.

*The adjusted variables included marital status, menopausal status, comorbidity score, histology, grade, use of chemotherapy, endocrine therapy, and radiation therapy*.

**Figure 4:**
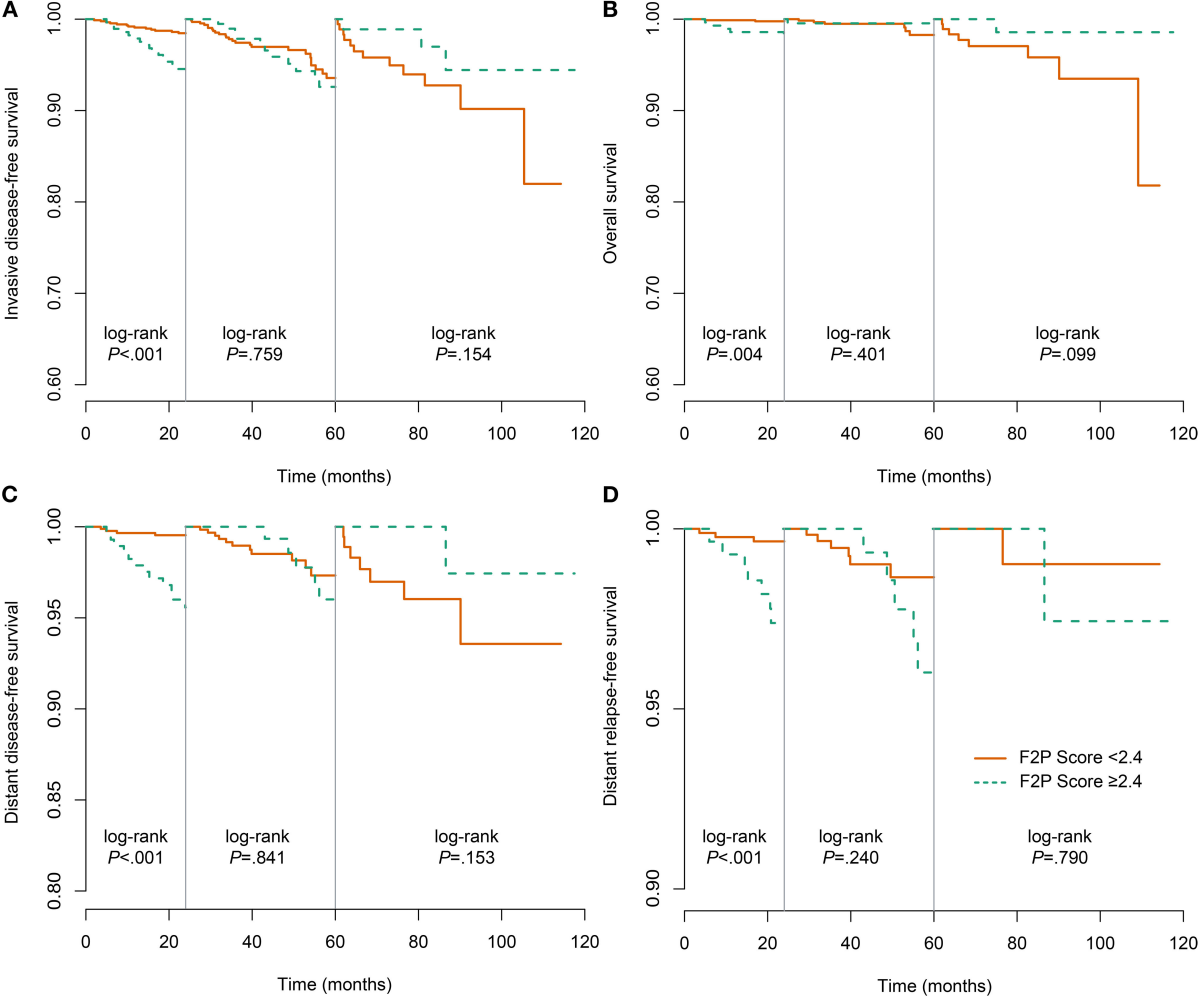
Landmark analysis of the association of F2P Score with different outcomes. **(A)** invasive disease-free survival; **(B)** overall survival; **(C)** distant disease-free survival; **(D)** distant relapse-free survival.

We also performed the analysis when the F2P score was adopted as a categorical predictor with a threshold of 2.4. A total of 873 (75.5%) patients were categorized as low risk (<2.4) and a small proportion (24.5%) were classified as high risk (≥2.4). The incidence rate of first-2-year relapse was 1.5% (95% CI, 0.7–2.4%) for patients at low risk while 6.3% (95% CI, 3.3–9.2%) for those of high risk (Log-rank *P* < 0.001) (Figure 4A and Table 5). After adjustment for clinicopathological characteristics, the categorical F2P Score also remained prognostic (HR, 3.68; 95% CI, 1.70–8.00; *P* = 0.001) (Table 4).

**Table 5:** Summary of end points.

**End point**	**F2P Score <2.4** **(N = 873)**		**F2P Score ≥ 2.4** **(N = 283)**
**Invasive disease-free survival (STEEP definition)**			
No. of patients with a first-2-year event (%)	13 (1.49)		17 (6.01)
2-year event-free rate (95% CI)	98.5 (97.6–99.3)		93.7 (90.8–96.7)
Log-rank *P*		<0.001	
**Invasive disease-free survival (excluding second primary**			
**non-breast cancer)**			
No. of patients with a first-2-year event (%)	9 (1.03)		16 (5.65)
2-year event-free rate (95% CI)	98.9 (98.3–99.6)		94.1 (91.4–97.0)
Log-rank *P*		<0.001	
**Distant disease-free survival (STEEP definition)**			
No. of patients with a first-2-year event (%)	4 (0.46)		12 (4.24)
2-year event-free rate (95% CI)	99.5 (99.1–100.0)		95.6 (93.2–98.1)
Log-rank *P*		<0.001	
**Distant relapse-free survival (excluding all-cause mortality)**			
No. of patients with a first-2-year event (%)	3 (0.34)		7 (2.47)
2-year event-free rate (95% CI)	99.7 (99.3–100.0)		97.4 (95.5–99.3)
Log-rank *P*		<0.001	
**Overall survival (STEEP definition)**			
No. of patients with a first-2-year event (%)	2 (0.23)		5 (1.77)
2-year event-free rate (95% CI)	99.8 (99.4–100.0)		98.2 (96.6–99.8)
Log-rank *P*		0.004	

STEEP, Standardized definitions for efficacy end points; CI, confidence interval.

*Therapy, and radiation therapy*.

As to other endpoints, the F2P Score also remained prognostic for 2-year DDFS, DRFS, and even OS when used as a continuous or categorical predictor (Tables 4, 5, Figures 4B–D).

## Discussion

Our study focused on very early relapse during the first two years after the initiation of endocrine therapy. We established an F2P Score, which is a novel prognostic approach to estimate the risk of first-2-year relapse in node-negative ER+/HER2– breast cancer by integrating both the clinicopathological and genomic factors. With per one unit increase in F2P score, an ~3-fold higher risk of first-2-year relapse was observed in the current study, indicating an increased potential of endocrine resistance. The prognostic value was also demonstrated across treatment subgroups, for example, in patients treated with TAM or AI and in those treated with chemotherapy or not. Likewise, the F2P Score may also quantify the likelihood of first-2-year relapse when employed as a categorical predictor.

Of special note is the fact that the continuous or categorical F2P Score may also estimate 2-year OS, since about half of the deaths occurred without relapse (4). It also correlates significantly with other different endpoints (DDFS/DRFS) when used as a continuous or categorical predictor.

Consistent with earlier findings, our study found that increased expression of estrogen-related genes (*ER, PGR*, and *BCL2*) and individual genes (*GSTM1* and *BAG1*) correlates with reduced likelihood of very early relapse, although most previous studies have focused on the years 0–5 (16, 29). Three genes of the scoring system came from the estrogen module of the 21-gene panel, supporting the idea that the F2P Score might indicate endocrine responsiveness. To date, numerous basic works have demonstrated that these estrogen-related genes play pivotal roles in the development of endocrine resistance (8–12). Zong et al. have also reported a higher rate of first-2-year relapse in patients with ER+/PR-/HER2– breast cancer when compared to those with ER+/PR+/HER2– tumor, suggesting that PR status could be adopted as a stable and reliable indicator of endocrine resistance in routine clinical practice (30). As to *CD68*, data at our disposal were interesting and we found that it played different roles between years 0–2 and 2–5, which was observed for *PGR* and *BAG1* as well. We hypothesize that this time-dependent effect could be related to different polarization patterns of tumor-associated macrophages because *CD68* is recognized as a pan-macrophage biomarker (31, 32). However, the 21-gene panel contains a small number of genes and thus, other important immunity-related genes could not be included in the analysis in the present study. The specific mechanism and whether the time-dependent effect is attributed to regulation of the immune system or not remains unclear, and further investigation is required.

Despite the potential prognostic value of these genes, it seems unlikely that molecular biomarkers alone can predict prognosis accurately, and thus, we combined gene expressions with clinicopathological characteristics. In clinical practice, age and tumor size are routinely adopted as indicators of relapse as well as to inform treatment decisions, and their combination with gene expression can improve the prognostic performance (5, 17–19). Pan et al. reported that patients with T2-stage tumors were at higher risk of distant recurrence when compared to those with T1-stage ones during adjuvant endocrine therapy (3). Consistently, the current study found that tumor size was also of strong predictive value for very early (first-2-year) relapse. Additionally, Dowsett et al. revealed that the integration of clinicopathological factors and RAM50 ROR could substantially enhance the prognostic ability of either clinicopathological or genomic approaches alone (17). Likewise, earlier studies also demonstrated improved performance when comparing 21-gene RS to its combination with clinicopathological variables (17, 19). Considering these existing data, the F2P Score, which integrated age, tumor size, and gene expressions, outperformed both gene-only models and several other prognostic tools in predicting first-2-year relapse. Another reason accounting for the superior performance may be that most prognostic tools were developed to estimate the risk of 5- or 10-year distant relapse rather than the first-2-year relapse examined in this discussion.

Very early relapse during the first two years of adjuvant endocrine therapy is regarded as an indicator of primary endocrine resistance (7). Accordingly, patients with a higher F2P Score might present low responsiveness to endocrine therapy and relatively unfavorable prognosis. Indeed, worse OS was observed for patients who experienced the first-2-year relapse in our study. These results are of particular clinical relevance and the F2P Score may inform decision making concerning the escalation in systemic therapy to improve the prognosis. In our study, patients receiving chemotherapy, aromatase inhibitors, or ovarian suppression presented a numerically lower HR, revealing the phenomenon that the association between the F2P Score and first-2-year relapse differs to a certain extent among patients with diverse treatment options. Consequently, patients with different F2P Scores may benefit from different systemic treatment approaches and the F2P Score may facilitate the treatment decision, but a cautious interpretation was required since there was no significant interaction. A wide confidence interval and limited events among a small number of cases using ovarian suppression were also observed. Consequently, further studies exploring the treatment strategy for patients with various F2P Scores are required.

Our work has several limitations. First, this is a retrospective study and thus, the F2P Score should be rigorously validated in prospective trials. Second, despite great necessity, external validation was not performed in our study since data presented herein came from a single institute. We performed the validation based on the bootstrap resampling method. Third, it is likely that some key genes might not have been detected due to the false negativity that results from a limited sample size. To address this issue, we included the genomic variables with *P* < 0.1. Fourth, data of gene expression in our study were from a 21-gene panel rather than the sequencing dataset, thus limiting the candidate genes selected for model development. Further investigation is required to establish a prognostic model based on a large microarray or sequencing dataset. However, it is also economic and of great convenience that patients who received a 21-gene test can also obtain an endocrine-sensitivity score. Last, a cutoff point of 2.5 for the F2P Score was determined. However, it is only for reference, since the gene expressions are tested based on various platforms and protocols in different institutes and standardization of the optimal cut-off point is required in a prospective setting.

In conclusion, the F2P Score reported herein, taking into account both the clinicopathological and genomic factors, may inform the prognosis and endocrine responsiveness in ER+/HER2– breast cancer. A higher F2P Score may indicate an increased likelihood of first-2-year relapse and therefore, suggest a potential resistance to endocrine therapy. Accordingly, after the potential benefits and risks have been considered, treatment escalation may be an alternative strategy for patients with a high F2P Score.

## Data Availability Statement

The datasets presented in this study can be found in online repositories. The names of the repository/repositories and accession number(s) can be found below: http://bcdb.mdt.team:8080/, Available on the request of the corresponding author.

## Ethics Statement

The current study was approved by the independent Ethical Committee of Ruijin Hospital. Written informed consent for participation was not required for this study in accordance with the national legislation and the institutional requirements.

## Author Contributions

CL, JW, and LZ: study concept and design. CL, JW, LL, and LZ: data analysis and interpretation. CL: visualization. LZ: final approval and funding acquisition. All authors: data acquisition and manuscript preparation.

## Conflict of Interest

The authors declare that the research was conducted in the absence of any commercial or financial relationships that could be construed as a potential conflict of interest.

## Abbreviations

ER: estrogen receptor
HER2: human epidermal growth factor receptor 2
PR: progesterone receptor
F2P: first-2-year prognosis
SJTU-BCDB: Shanghai Jiao Tong University Breast Cancer Database
IDFS: invasive disease-free survival
DDFS: distant disease-free survival
DRFS: distant relapse-free survival
OS: overall survival
HR: hazard ratio
CI: confidence interval
C-index: Harrell's concordance index
BIC: Bayesian information criteria
NRI: net reclassification index
LR-χ^2^: likelihood ratio χ^2^
IHC: immunohistochemistry
CTS: clinical treatment score
RT-PCR: reverse transcriptase–polymerase chain reactions
TAM: tumor-associated macrophages.
