# Supplementary figures and images for: A Novel Prognostic Scoring System Integrating Gene Expressions and Clinicopathological Characteristics to Predict Very Early Relapse in Node-Negative Estrogen Receptor-Positive/HER2-Negative Breast Cancer
Source: Front Oncol. 2020 Sep 11;10:1335. doi: 10.3389/fonc.2020.01335 (PMC7518385; doi:10.3389/fonc.2020.01335)

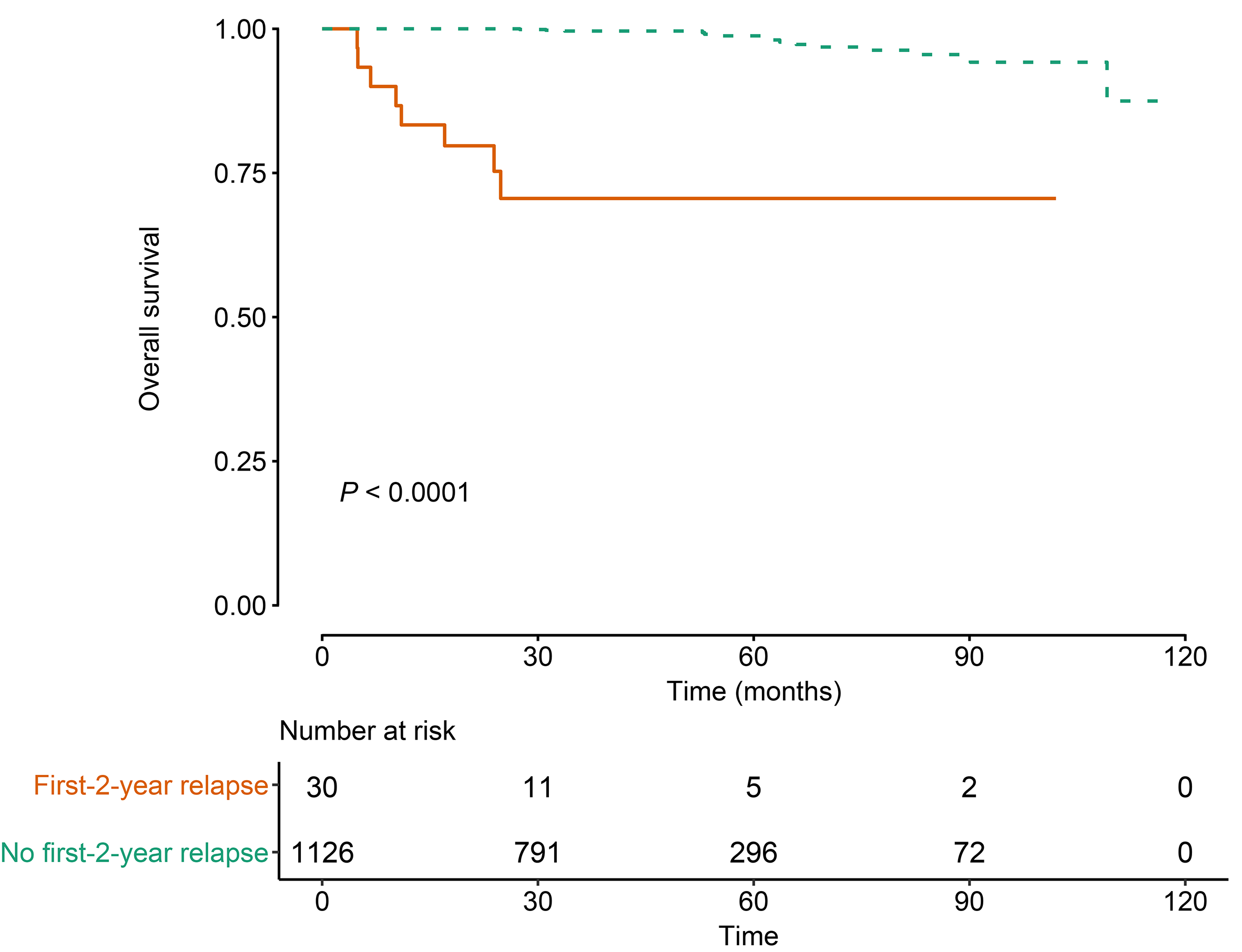

Supplement: Supplementary file 1 [file Image_1.TIF]
