# Supplementary material for: A Novel Prognostic Scoring System Integrating Gene Expressions and Clinicopathological Characteristics to Predict Very Early Relapse in Node-Negative Estrogen Receptor-Positive/HER2-Negative Breast Cancer
Source: Front Oncol. 2020 Sep 11;10:1335. doi: 10.3389/fonc.2020.01335 (PMC7518385; doi:10.3389/fonc.2020.01335)
Supplement: Supplementary file 2 [file Table_1.DOCX]

**Table S1. Cox regression analysis revealing the association of clinicopathological and genomic variables with invasive disease-free survival according to time periods of follow-up**

|  | **0-5 years** |  |  | **0-2 years** |  |  | **2-5 years** |  |  | **Interaction *P*** |
| --- | --- | --- | --- | --- | --- | --- | --- | --- | --- | --- |
|  | **HR 95% CI** | ***P*** |  | **HR 95% CI** | ***P*** |  | **HR 95% CI** | ***P*** |  |  |
| ***Part I: Clinical variables*** | | | | | | | | | | |
| Age, vs ≤ 50 years | 0.75 (0.46-1.23) | .255 |  | 0.51 (0.25-1.05) | .068 |  | 1.05 (0.52-2.14) | .886 |  | .163 |
| Menopausal status,  vs postmenopausal | 1.01 (0.61-1.66) | .967 |  | 1.30 (0.63-2.68) | .472 |  | 0.81 (0.40-1.62) | .550 |  | .351 |
| Comorbidity score |  |  |  |  |  |  |  |  |  |  |
| 1 vs 0 | 0.83 (0.33-2.09) | .698 |  | 1.51 (0.52-4.40) | .450 |  | 0.30 (0.04-2.21) | .237 |  | .162 |
| ≥2 vs 0 | 2.20 (1.15-4.24) | .018 |  | 2.57 (0.97-6.81) | .058 |  | 1.96 (0.81-4.72) | .135 |  | .684 |
| Histology |  |  |  |  |  |  |  |  |  |  |
| ILC vs IDC | 1.10 (0.34-3.51) | .873 |  | 0.87 (0.12-6.44) | .893 |  | 1.26 (0.30-5.27) | .751 |  | .769 |
| Others vs IDC | 1.23 (0.58-2.59) | .580 |  | 1.82 (0.70-4.78) | .222 |  | 0.80 (0.24-2.62) | .710 |  | .290 |
| Grade |  |  |  |  |  |  |  |  |  |  |
| Moderately vs. well | 1.00 (0.39-2.58) | .994 |  | 2.50 (0.33-19.01) | .376 |  | 0.62 (0.21-1.87) | .398 |  | .237 |
| Poorly vs. well | 1.66 (0.62-4.44) | .315 |  | 3.77 (0.47-30.18) | .211 |  | 1.12 (0.36-3.52) | .848 |  | .315 |
| Tumor size, vs ≤ 2cm | 2.73 (1.68-4.43) | <.001 |  | 4.15 (1.98-8.72) | <.001 |  | 1.94 (1.00-3.76) | .050 |  | .134 |
| PR status,  vs PR-positive | 1.68 (0.95-2.99) | .077 |  | 2.06 (0.92-4.62) | .081 |  | 1.39 (0.61-3.18) | .432 |  | .509 |
| Ki 67, vs < 20% | 1.56 (0.96-2.52) | .072 |  | 1.14 (0.56-2.35) | .717 |  | 2.02 (1.04-3.89) | .037 |  | .255 |
| ***Part II: Genomic variables*** | | | | | | | | | | |
| Estrogen module |  |  |  |  |  |  |  |  |  |  |
| ESR1 | 0.90 (0.77-1.04) | .154 |  | 0.77 (0.62-0.96) | .017 |  | 1.01 (0.83-1.25) | .889 |  | .071 |
| PGR | 0.93 (0.84-1.04) | .201 |  | 0.83 (0.71-0.96) | .015 |  | 1.04 (0.90-1.21) | .574 |  | .033 |
| BCL2 | 0.83 (0.67-1.04) | .099 |  | 0.75 (0.54-1.03) | .075 |  | 0.90 (0.67-1.22) | .517 |  | .395 |
| SCUBE2 | 0.93 (0.82-1.06) | .282 |  | 0.93 (0.77-1.12) | .445 |  | 0.93 (0.79-1.11) | .448 |  | .953 |
| Proliferation module |  |  |  |  |  |  |  |  |  |  |
| Ki67 | 1.23 (0.99-1.54) | .060 |  | 1.35 (0.96-1.89) | .102 |  | 1.16 (0.87-1.54) | .323 |  | .489 |
| STK15 | 1.13 (0.94-1.36) | .184 |  | 1.05(0.78-1.40) | .761 |  | 1.19 (0.95-1.50) | .137 |  | .494 |
| Survivin | 1.09 (0.90-1.31) | .364 |  | 1.10 (0.82-1.48) | .532 |  | 1.08 (0.85-1.38) | .508 |  | .944 |
| CCNB1 | 1.03 (0.83-1.28) | .785 |  | 0.85 (0.61-1.17) | .309 |  | 1.20 (0.90-1.58) | .209 |  | .112 |
| MYBL2 | 1.15 (0.95-1.39) | .160 |  | 1.11 (0.82-1.51) | .489 |  | 1.17 (0.92-1.49) | .211 |  | .807 |
| Invasion module |  |  |  |  |  |  |  |  |  |  |
| MMP11 | 1.10 (0.94-1.29) | .253 |  | 0.98 (0.77-1.25) | .884 |  | 1.19 (0.96-1.48) | .107 |  | .234 |
| CTSL2 | 1.08 (0.89-1.30) | .424 |  | 1.18 (0.88-1.58) | .264 |  | 1.02 (0.79-1.30) | .906 |  | .440 |
| HER2 module |  |  |  |  |  |  |  |  |  |  |
| GRB7 | 1.11 (0.87-1.41) | .390 |  | 0.86 (0.60-1.23) | .414 |  | 1.33 (0.98-1.80) | .100 |  | .103 |
| HER2 | 0.94 (0.76-1.15) | .553 |  | 0.88 (0.64-1.20) | .404 |  | 0.99 (0.75-1.30) | .946 |  | .562 |
| GSTM1 | 0.84 (0.69-1.02) | .086 |  | 0.80 (0.59-1.06) | .092 |  | 0.87 (0.67-1.14) | .315 |  | .663 |
| CD68 | 0.93 (0.73-1.18) | .549 |  | 0.63 (0.46-0.88) | .006 |  | 1.27 (0.93-1.74) | .129 |  | .002 |
| BAG1 | 0.99 (0.79-1.27) | .967 |  | 0.73 (0.51-1.04) | .081 |  | 1.26 (0.92-1.71) | .154 |  | .024 |

Abbreviations: IDC, infiltrating ductal carcinoma; ILC, infiltrating lobular carcinoma; PR, progesterone receptor; HR, hazard ratio; CI, confidence interval.
